# A Plasma Transmitting Source for Borehole Acoustic Reflection Imaging

**DOI:** 10.3390/s22208050

**Published:** 2022-10-21

**Authors:** Xiaolong Hao, Jing Zhou, Haiyan Shang, Haiming Xie, Wei Wang, Cheng Yang

**Affiliations:** 1 Downhole Measurement & Control Research Department, National Engineering Laboratory of Petroleum Drilling Technology, Xi’an Shiyou University, Xi’an 710065, China; 2 School of Electronic Engineering, Xi’an Shiyou University, Xi’an 710065, China

**Keywords:** acoustic reflection imaging, plasma source, acoustic-electrical characteristics, directional radiation, detection depth, high resolution

## Abstract

The detection depth of current borehole acoustic reflection imaging is only tens of meters without high resolution. This considerably limits its wide application in the identification and fine description of unconventional reservoirs and in the optimization of drilling trajectories. Increasing the directional energy from the transmitter to a geological structure is an excellent way to solve this issue. In this study, a plasma source with a parabolic reflector was introduced during borehole acoustic reflection imaging. First, an experimental system was built for testing the plasma source. Next, the acoustic-electrical characteristics and directional radiation of the source were studied using experiments and a numerical simulation. Finally, the advantages, disadvantages, and feasibility of the plasma-transmitting source were analyzed; some suggestions for further work on the source and its logging application were proposed. The experimental and simulation results show that the use of a plasma source with a parabolic reflector can increase the detection depth of borehole acoustic reflection imaging to hundreds of meters with high resolution. This is crucial in imaging the geological structures near boreholes and enhancing oil–gas exploration and development.

## 1. Introduction

Obtaining accurate formation information in large three-dimensional (3D) spaces around boreholes will aid in improving the efficiency of oil–gas exploration and development. Borehole acoustic reflection imaging is a rapidly developing technology that can detect geological structures within tens of meters or more of a borehole. It can fill the gap between the shallow detection of conventional acoustic logging and the low resolution of seismic exploration. This logging method has the obvious advantage of describing the lateral inhomogeneity in carbonate reservoirs, the attitudes and distributions of fractures, and small geological structures near boreholes [1,2]. Therefore, it has been widely used in the identification and fine description of unconventional reservoirs and in the optimization of drilling trajectories [3,4].

However, current tools work at low detection depths and cannot provide satisfactory resolutions simultaneously, which seriously affects their application performance. This is attributed to the transmitter power, receiver sensitivity, formation attenuation, and other factors. Increasing the directional energy from the transmitter to a geological structure is an effective way to address this limitation. Currently, acoustic transducers based on the piezoelectric effect are used in various fields [5,6,7]. Three sources are primarily used in the acoustic transmitter: monopole, dipole, and phased-array sources. Many theoretical analyses, numerical simulations, experimental tests, and field applications have been conducted to develop acoustic reflection imaging based on these sources [7,8,9].

These three sources have certain advantages and disadvantages regarding source power, borehole effect, radiation directivity, formation attenuation, and radial resolution. A piezoelectric monopole source generally works in the range of 10–20 kHz and generates a wavefield without directivity in a homogeneous medium. It has high power, and its sound-pressure level (SPL) 1 m from the source in water can reach ~210 dB [10]. Based on the source and a monopole receiver, early tools can only determine the distance from a reflector to a borehole and cannot determine the specific azimuth [11]. Currently, such tools use azimuth-array receivers to improve the azimuthal resolution [12]. A dipole source generates a flexural wave and works at ~4 kHz. It has medium power and a dumbbell-shaped wavefield in a homogeneous medium. Based on a cross-dipole logging tool, the azimuth and distance of a geological reflector with respect to the borehole can be determined simultaneously, although the azimuth has 180° of uncertainty [13]. The shear horizontal wave has a wider radiation coverage and higher reflection sensitivity than the shear vertical wave [14,15]. The latest shear-wave reflection imagers, such as the VMSI tool, can determine reflectors 80 m away from a borehole [16]. A phased-array source can be linear, arc, or combined. A combined array is formed by several arc arrays that are placed equally along the tool axis. By controlling the excitation-delay times of the array elements, the main lobe of an acoustic beam can be deflected vertically and horizontally to achieve a 3D directional radiation [17]. A linear array changes the vertical directivity of an acoustic beam, while an arc array affects the horizontal directivity. The arc-array source has been combined with an azimuth-array receiver to evaluate the cementing quality azimuthally in a cased hole [18]. However, owing to the limited tool space, the energy from the arc-array source is too small to be widely used in borehole acoustic reflection imaging. The tool based on a monopole linear-array source can detect geological structures ~40 m away from the borehole with an azimuth resolution of 22.5° [19].

However, it cannot satisfy the demand of the next-generation tool regarding high-resolution imaging at a detection depth of hundreds of meters, particularly in directional energy and frequency coverage. Therefore, developing a new downhole transmitting source with high power, wide band, and excellent directional radiation is necessary to achieve deep detection with high resolution in borehole acoustic reflection imaging.

The plasma is composed of ions, electrons and unionized neutral particles. It is widely present in gas, metal, semiconductor and electrolyte solutions. There are two kinds of plasma: high-temperature plasma and low-temperature plasma. Chandra et al. used an quantum hydrodynamic model to study the quantum plasma based on a Hometopy Assisted Symbolic Simulation technique and INSAT-FORK code [20,21,22]. A plasma source in conductive liquid is based on the electrohydraulic effect and uses a high power supply to discharge a dielectric load in the electrode gap with high voltage and current. Researchers have investigated the micromechanism and macroperformance of underwater arcs and corona discharges, such as the dynamic evolvement process of bubbles, the features and influential factors of shock waves and source-directional radiation [23,24]. The models of thermal processes and time-varying resistance can be used to theoretically analyze arc discharge [25]. The discharge power, electrode spacing, temperature (0–90 °C), conductive fluids (e.g., tap water, salt water, and oil–water mixture), hydrostatic pressure (0–60 MPa) and other factors determine whether electric breakdown occurs and considerably affect the characteristics of the generated shock waves [24,26,27,28,29]. The near-field propagation of an arc discharge can be simulated using a cylindrical-source model with a high length-to-diameter ratio. When the propagation distance is 10 times longer than the electrode spacing, the cylindrical wave becomes spherical [30]. The peak pressure of the shock wave can increase by >20 dB and the acoustic field can change to different directivities when using a parabolic reflector [31]. The source has been used in underwater warfare, seismic exploration, oil–gas-plug removal and fracturing [32,33]. Sun et al. completed a cross-hole seismic exploration with a 1210-m depth and 204-m spacing [34,35]. They used a source with a capacitance of 567 µF and an energy of 6.8 kJ when the voltage was 4900 V. Yan et al. developed a reservoir plug-removal technology based on shock waves [33]. When the source had an energy of 3 kJ (20 kV and 15 µF), the SPL measured 1 m away from the source in water reached 255 dB. The frequency components of the generated shock wave ranged from 0 to 100 kHz. 

Based on the abovementioned work, a plasma-transmitting source was introduced in borehole acoustic reflection imaging in this study. An experimental system was built to evaluate the plasma source. The acoustic–electrical characteristics and the directional radiation of the source were studied through experiments and numerical simulations. The feasibility of the plasma-transmitting source was analyzed and some suggestions for further work on the source and its logging application are proposed.

## 2. Experimental System

**Figure 1:**
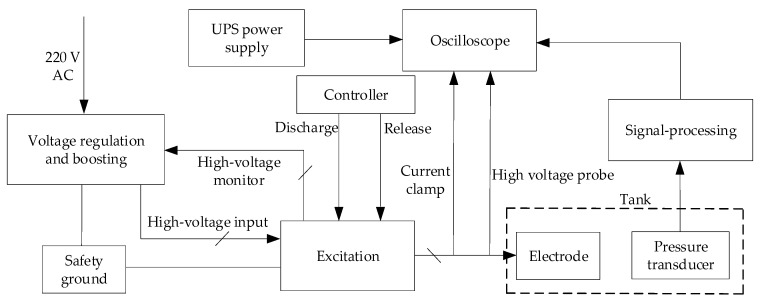
Experimental system for the plasma source.

An experimental system was built to provide working conditions for the plasma source and measure its output. Figure 1 shows the operation of the system, which mainly includes an electrode, a pressure transducer, a tank, an oscilloscope, and three circuit modules (voltage regulation and boosting, electrode excitation, and signal processing). To perform the tests safely, the voltage regulation and boosting module and excitation module uses 220 V of power in alternating current (AC) and is connected to the safety ground, whereas the oscilloscope and signal-processing module are powered by an uninterruptible power supply. 

The voltage-regulation-and-boosting module comprises a regulator and a booster. The 220-Volt AC input is transformed into an adjustable signal from 0 to 250 V via the regulator. The booster increases this signal to thousands of volts, outputs it to the excitation module and monitors its change.

**Figure 2:**
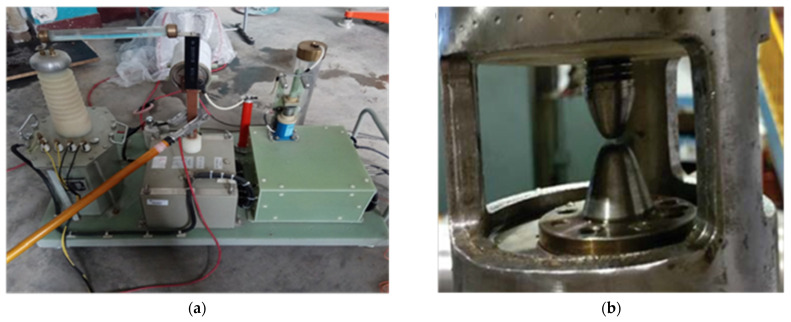
Actual photographs of (**a**) excitation module and (**b**) electrode module.

The excitation module includes a charging, a discharging, and a release submodule. Figure 2a shows an actual photograph of the module. The charging submodule primarily comprises a 15-microfarad storage capacitor, a high-voltage silicon stack, and a current-limiting resistor to store energy for the source excitation. The electrode module is excited by the discharge submodule using a discharge switch in the controller. This can cause an electrohydraulic effect. The residual energy after excitation can be released by the release switch to ensure each operation’s independence. Furthermore, if the discharging submodule cannot works normally, the release submodule can forcibly release the high energy in the storage capacitor to ensure safety in testing.

The electrode module was designed as a cylinder with several acoustic windows to adapt to the borehole environment and acoustic logging tool. The metal electrode is at the center of the cylinder. Figure 2b shows an actual photograph of the electrode at a specific distance. The electrode module and pressure transducer are immersed in a tank containing liquid and the distance between them is adjustable. The electrode module generates an acoustic signal; the pressure transducer receives the acoustic signal and converts it into an electrical signal. This electrical signal is processed and then uploaded to the oscilloscope via the signal-processing module. Furthermore, the electrode voltage and current changes during source excitation are monitored using a high-voltage probe and a current clamp.

A high-voltage pulse is output to the electrode immediately after the discharge switch is turned on, causing high field strength in the electrode gap and generating an electric arc. Additionally, the arc expands rapidly and produces a shock wave that spreads outward because of the weak liquid compressibility. This is the working mechanism of a plasma source and the foundation of the design of the experimental system.

The system can output 0–25 kV in adjustable voltage to the electrode. The measuring range of the high-voltage probe and current clamp are 0–40 kV and 0–25 kA, respectively. The pressure transducer can measure up to 300 MPa with a sensitivity of 0.73 mV/kPa.

## 3. Experimental Test of Acoustic and Electrical Characteristics

Based on the experimental system, the acoustic and electrical characteristics of a plasma source were tested according to the following procedure. First, turn on the charging switch to charge the storage capacitor. Next, turn on the discharge switch to output a high voltage to the electrode to generate a shock wave. Finally, use the release switch to release the residual energy. The electrical characteristics of the plasma source were evaluated by measuring the electrode voltage and current changes. Its acoustic characteristics was evaluated using the acoustic signal received by the pressure transducer.

### 3.1. Electrical Characteristics

**Figure 3:**
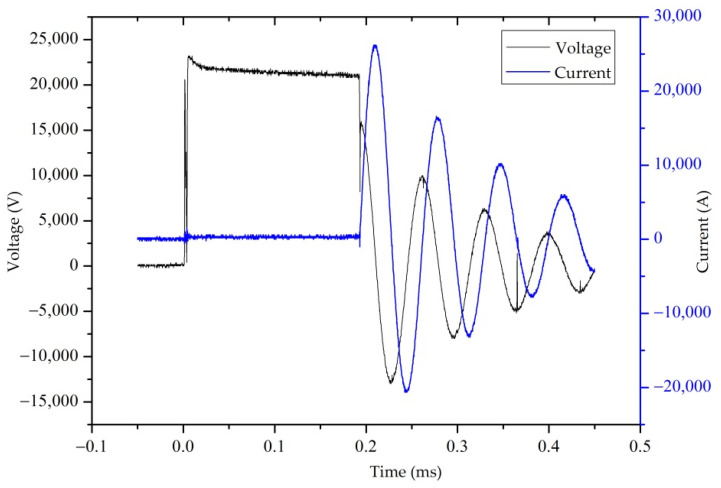
Voltage and current changes during source excitation.

Figure 3 shows a double-Y diagram of the electrode voltage and current changes for the time during the source excitation. The electrode voltage changes to 22 kV immediately once the discharge switch is turned on. At this excitation voltage, the prebreakdown process takes ~0.19 ms and the formation time of a plasma channel is ~5 μs. After this stage, the electrode voltage decreases as oscillation attenuation. The peak current reaches 25 kA and attenuates for several periods.

### 3.2. Acoustic Characteristics

**Figure 4:**
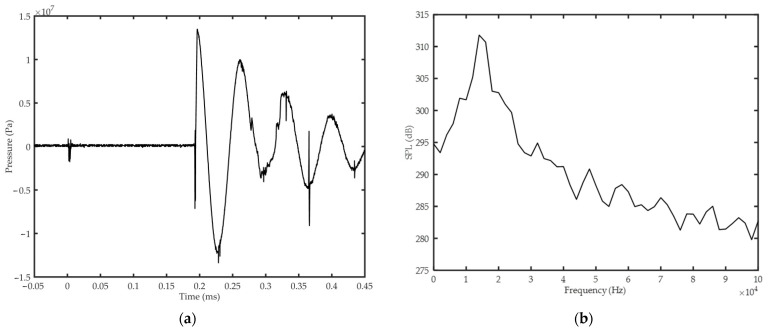
Variations of (**a**) acoustic pressure with time and (**b**) SPL with frequency.

Figure 4a,b shows the acoustic pressure and SPL variations with respect to time and frequency, respectively. The pressure was measured 0.16 m away from the source under the abovementioned excitation conditions. Figure 4a shows that the maximum acoustic pressure reaches 13 MPa. As shown in Figure 4b, the source has a high SPL ranging from 0 to 40 kHz and the SPL exceeds 310 dB at 14 kHz. This significantly exceeds the 210 dB from the monopole source. These data confirm that the plasma source is a high-power, wide-band source.

## 4. Design of Directional Radiation

**Figure 5:**
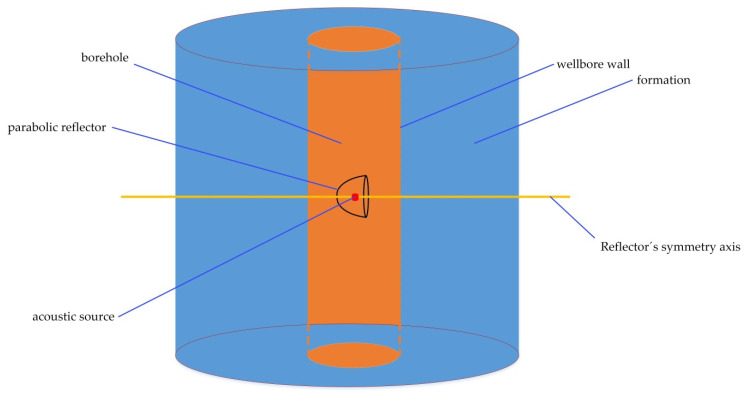
Diagram of the acoustic source with a parabolic reflector in the logging environment.

**Figure 6:**
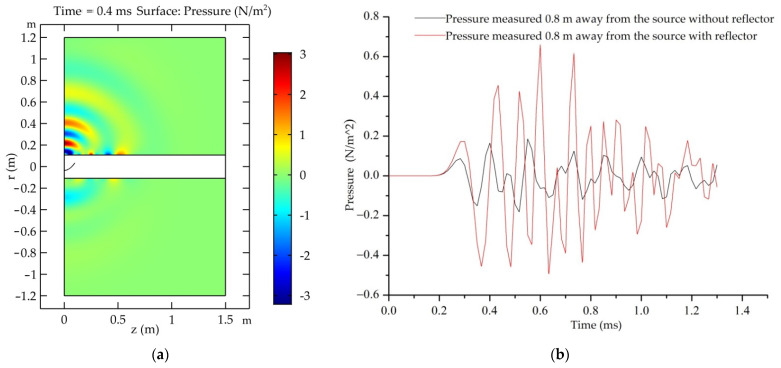
(**a**) Wavefield snapshot at *t* = 0.4 ms and (**b**) pressure–gain effect due to the parabolic reflector.

A parabolic reflector can be used to improve the directional radiation of a plasma source in borehole acoustic reflection logging. Figure 5 shows a diagram of the acoustic source with a parabolic reflector in the logging environment. The whole space includes two parts: the formation and the cylindrical wellbore. The parabolic reflector is located in the borehole and the acoustic source is fixed at its focus. The whole space and parabolic reflector have a common axis of rotation symmetry in a small range. Therefore, a two-dimensional axisymmetric model, as shown in Figure 6a, was established to numerically simulate the wavefield change due to a parabolic reflector in the formation with a borehole. The borehole has a diameter of 0.216 m and the filled water has a density of 1000 kg/m^3^ and a P-wave velocity of 1500 m/s. The reflector and its position can be described by *z*^2^ = 0.14 (*r* + 0.035), where *z* represents the well-axis direction and *r* is its radial direction with −0.035 m ≤ *r* ≤ 0.035 m. The source is a Ricker wavelet with a dominant frequency of 10 kHz. The sandstone in the formation has a density, a P-wave velocity and an S-wave velocity of 2650 kg/m^3^, 3800 m/s, and 2300 m/s, respectively.

A pressure–acoustics interface was used for the borehole zone and the solid mechanics interface was used for the formation [36]. The wave equation in the fluid is shown in Equation (1), where $\rho_{f}$, $c_{f}$, *p* are the liquid density, velocity and pressure, respectively. The wave equation in the solid is shown in Equation (2), where ρ, ***u***, σ  are the solid density, displacement and stress, respectively. The normal displacement and stress at the wellbore wall are continuous. The boundaries of the water, the formation and the inner wall of the reflector were addressed using plane-wave radiation, a low-reflecting boundary and a hard acoustic boundary, respectively.

(1) $$\frac{1}{\rho_{f}c_{f}{}^{2}}\frac{\partial^{2}p}{\partial t^{2}}+\nabla \cdot \left(-\frac{1}{\rho_{f}}\nabla p\right)=0$$

(2) $$\rho \frac{\partial^{2}\mu }{\partial t^{2}}-\nabla \cdot \sigma =0$$

Figure 6a shows the wavefield snapshot at time *t* = 0.4 ms. The energy is concentrated at a narrow angle (~60°) near the reflector’s symmetry axis. This is much better than the case in which the source does not use a reflector and has no evident radiation directivity. Figure 6b shows the pressure–gain effect on the shock wave measured 0.8 m away from the source at the abovementioned symmetry axis. When using the parabolic reflector, the peak pressure can increase by >10 dB. These results demonstrate that the plasma source with a parabolic reflector can achieve good directional radiation and energy enhancement.

## 5. Application of a Plasma Source in Borehole Acoustic Reflection Imaging

The application of a plasma-transmitting source in borehole acoustic reflection logging was analyzed from the following two perspectives: (1) application feasibility and (2) further work on the source and its logging application.

### 5.1. Feasibility of a Plasma-Transmitting Source

This research demonstrates that a plasma source with a parabolic reflector can release significant energy, generate strong wide-band shock waves, and achieve good directional radiation. These results indicate that plasma sources might play a crucial role in borehole acoustic reflection imaging.

The feasibility of using the plasma source as a logging transmitter was compared with the current sources in Table 1 for further analysis. Monopole, dipole and phased-array sources have advantages and disadvantages regarding source power, formation attenuation, radiation directivity and radial resolution. The plasma source has the largest power, and the generated shock waves contain high- and low-frequency components. The high-frequency components have excellent radial resolutions, large attenuations and shallow detection depths, while the low-frequency components have the opposite features. Therefore, acoustic reflection imaging based on a plasma source has the advantage of simultaneously achieving a deep detection in the far-borehole zone and a high-resolution detection near the borehole. Furthermore, the source has a small-volume electrode and is not limited by the tool space. A tool based on the plasma source can achieve good azimuthal resolution by designing a directional transmitter and adopting an azimuth-array receiver. Therefore, applying a plasma-transmitting source in borehole acoustic reflection imaging is feasible and promising.

### 5.2. Further Work on the Source and Its Logging Application

Although feasible and promising, borehole acoustic reflection imaging based on a plasma source is still in its early stage. Considerable research with theoretical analyses, numerical simulations, experimental tests and field applications is required to introduce this source into logging applications, including the following aspects:

(1) The influencing factors and control methods for the acoustic and electrical characteristics of the plasma source. It is necessary to further investigate the influence of different electrical parameters (e.g., discharge voltage and storage capacitance), electrode systems (e.g., structure and spacing) and working environments (e.g., dielectric conductivity, temperature and static pressure) on the source characteristics. In particular, the source performance in a logging environment with high temperature (>125 °C), high pressure (>100 MPa) and liquid-filled borehole needs to be studied.
(2) The directional radiation of the source. The near-field characteristics of the source must be investigated. It is necessary to manufacture parabolic reflectors with different parameters and evaluate them experimentally according to the numerical simulation. A small-sized controllable rotating parabolic reflector should be considered to achieve directional and scanning radiation. This could not only enhance the directional energy towards the target but also improve the azimuthal resolution of the tool based on the source.
(3) The acoustic reflection imaging response of a geological structure. The logging response of a geological structure with different parameters (e.g., formation, distance and attitude) must be considered to provide a foundation for tool design and logging inversion based on plasma sources.
(4) Downhole realization. A small downhole plasma source should be developed to prevent the energy losses caused by wireline transmission. Considerable work must be conducted, e.g., optimizing the electrode structure to improve its stability, selecting high-temperature-resistant components and determining a suitable level of directional-radiation energy to ensure deep detection without damaging the borehole.
(5) Data acquisition and processing based on the source. In terms of data acquisition, two points need to be considered: (1) increase the acquisition time of the full waveform to correspond with the increased detection depth and (2) separate the detection process for the near- and far-borehole zones into two data acquisitions. This helps to improve the signal-to-noise ratio of the reflection wave. In terms of data processing, the extraction of the weak reflection wave and corresponding high-resolution imaging need further research.

## 6. Conclusions

Based on the investigation of the existing sources, a plasma source was introduced into borehole acoustic reflection imaging. An experimental system was built for evaluating the plasma source, and the source’s acoustic–electrical characteristics and directional radiation were studied through experiments and a numerical simulation. The feasibility of the plasma transmitting source was analyzed and some suggestions for further work on the source and its logging application were proposed. The following conclusions can be drawn.

(1) The current monopole, dipole and phased-array sources cannot satisfy the demand of next-generation tools regarding high-resolution imaging at detection depths of hundreds of meters, particularly with respect to the effective energy and frequency coverage.
(2) A plasma source with a parabolic reflector can release high levels of energy, generate strong wide-band shock waves and achieve good directional radiation. The development of borehole acoustic reflection imaging based on this source is feasible and promising. This would considerably increase the detection depth with high resolution to improve the performance of oil–gas exploration and development.
(3) Borehole acoustic reflection imaging based on plasma sources is still in the early stage and requires further research in terms of theoretical analyses, numerical simulation, experimental testing and field applications.

## Figures and Tables

**Table 1:** Borehole acoustic reflection imaging techniques based on different sources.

Source Type	Plasma	Monopole	Dipole	Phased Arc Array
Power	higher (255 dB at 1 m in water)	high (210 dB at 1 m in water)	medium	low
Frequency	0–100 kHz, adjustable	10–20 kHz	2–5 kHz	about 14 kHz
Formation attenuation	frequency dependent, small-large	medium-large	small	medium
Radiation directivity	designable	no	dumbbell shape	directional enhancement
Detection depth	deeper (>100 m)	deep	deep (tens of meters)	shallow (several meters)
Azimuthal resolution with azimuth-array receiver	designable, better than monopole	medium (22.5°)	good, uneven	excellent
Radial resolution	frequency dependent, poor-excellent	good-excellent	poor	good
Disadvantage	physical mechanism and influencing factors are complex, relevant research is in the early stage	no radiation directivity, medium azimuth resolution, large attenuation	uneven azimuth resolution and 180° uncertainty, poor radial resolution	low power, large attenuation, shallow detection depth

## Data Availability

Not applicable.
